# Dynamics and Cell-Type Specificity of the DNA Double-Strand Break Repair Protein RecN in the Developmental Cyanobacterium *Anabaena* sp. Strain PCC 7120

**DOI:** 10.1371/journal.pone.0139362

**Published:** 2015-10-02

**Authors:** Sheng Hu, Jinglan Wang, Li Wang, Cheng-Cai Zhang, Wen-Li Chen

**Affiliations:** 1 State Key Laboratory of Agricultural Microbiology, Huazhong Agricultural University, 430070 Wuhan, China; 2 Aix-Marseille Université and Laboratoire de Chimie Bactérienne (UMR7283), 31 Chemin Joseph Aiguier, 13402 Marseille cedex 20, France; Centre National de la Recherche Scientifique, Aix-Marseille Université, FRANCE

## Abstract

DNA replication and repair are two fundamental processes required in life proliferation and cellular defense and some common proteins are involved in both processes. The filamentous cyanobacterium *Anabaena* sp. strain PCC 7120 is capable of forming heterocysts for N_2_ fixation in the absence of a combined-nitrogen source. This developmental process is intimately linked to cell cycle control. In this study, we investigated the localization of the DNA double-strand break repair protein RecN during key cellular events, such as chromosome damaging, cell division, and heterocyst differentiation. Treatment by a drug causing DNA double-strand breaks (DSBs) induced reorganization of the RecN focus preferentially towards the mid-cell position. RecN-GFP was absent in most mature heterocysts. Furthermore, our results showed that HetR, a central player in heterocyst development, was involved in the proper positioning and distribution of RecN-GFP. These results showed the dynamics of RecN in DSB repair and suggested a differential regulation of DNA DSB repair in vegetative cell and heterocysts. The absence of RecN in mature heterocysts is compatible with the terminal nature of these cells.

## Introduction

DNA repairing is essential for keeping the integrity of chromosome for cell survival. DNA double-strand breaks (DSBs), including one-ended DSBs and two-ended DSBs can be repaired by homologous recombination (HR), whereas two-ended DSBs can also be repaired by nonhomologous end-joining or single-strand annealing. In recent years, more and more gained details have provided a better understanding of this stepwise process in bacteria [1–5], and the advance of fluorescent tracking technology has allowed the related proteins to be directly visualized in situ [6–8]. However, the understanding of the DNA DSB repairing process is not very clear.

When DNA damage occurs, complex molecular machinery involved in DNA repair is recruited at the site of the damage and cell proliferation is arrested. RecN is one the first elements that respond to these damages [9,10]. RecN together with RecA, RecF, RecO, and other elements form a repair center at the site of DNA damage [11,12]. RecN originally identified in *Escherichia coli* exists in most bacteria. It is a cohesin-like protein [13] and belongs to the SMC (structural maintenance of chromosome) protein family. RecN proteins of bacteria appear as typical ABC-type proteins with two walker domains and associated signature sequences, and it is conserved in length and functional motifs [14]. *recN* mutants are more sensitive to chromosome damaging caused by mitomycin C (MMC), UV or γ-irradiation in *E*. *coli* [15,16], thus leading to the accumulation of DNA DSBs, chromosomal rearrangements or deletions [15,17]. RecN is localized as a nucleoid-associated focus in a majority of cells when the chromosome damage is induced [12,18]. In *Bacillus subtilis*, RecN, in concert with the polynucleotide phosphorylase (PNPase), interacts with the 3’-OH of the damaged sites to form a repair center and catalyzes the basal end-resection of the 3’-end of single stranded DNA (ssDNA) molecules in the presence of Mn^2+^ and ATP [7,19,20]. Upon exposure to MMC or X-ray radiation, random DSBs occur at the chromosome of *B*. *subtilis*, and cell division and growth will be arrested; in response to such damages, RecN assembles within 15–30 min as a single focus in the cells [12]. The foci are usually found at the center and are thought to correspond to the ‘replication factory’ of the cells [21]. RecN-GFP foci are rarely observed in the absence of DNA damage, while SbcC-GFP foci are always present throughout the whole cell cycle [22,23]. In the presence of DNA damage, SbcC foci are mostly localized within the nucleoid at itscenter, where the DNA polymerase complex is usually localized; in contrast, RecN forms mainly one focus at any location at the nucleoids [12,22].

*Anabaena* sp. strain PCC 7120 (hereafter *Anabaena*) is a filamentous cyanobacterium usually used as a model in the study of cellular differentiation and multicellular pattern formation. When deprived of combined nitrogen, *Anabaena* develops heterocyst in 24 h specifically for fixing N_2_. In each filament, about 5%~10% of vegetative cells would differentiate into heterocysts distributed in a semi-regular pattern. The process of heterocyst differentiation is mediated by complicated mechanisms [24,25]. *Anabaena* may contain more than 10 copies of the chromosome [26]. Some previous works reported that cell division cycle might be involved in heterocyst differentiation [27–29]. DNA damage could cause replication fork to collapse and the cell cycle to stop and it is unable to divide heterocysts since they were terminally differentiated cells. However, some DNA polymerases, such as polymerase III, are present in heterocysts [30].

To examine how DNA repair operates in cyanobacteria with terminal differentiation, we focused on the RecN of *Anabaena*. We examined the behaviors of RecN *in vivo* by using a RecN-GFP fusion driven by its native promoter. We proved that RecN molecules were organized into focal assemblies that moved dynamically within the cells. When the damage was enhanced in vegetative cells, RecN moved towards the center of the cells. However, RecN is absent in mature heterocysts.

## Materials and Methods

### Bacterial strains and manipulation

Strains and plasmids used in this study were described in Supporting Information (S1 Table). *Anabaena* strains were cultured in BG11 or BG11_0_ media [28,31–33]. *E*. *coli* strains were cultured according to previously described method [33]. Conjugation and heterocyst enrichment were carried out according to previous methods [34–36].

### Plasmid preparation

The *E*. *coli* strain used in cloning experiments was DH5α. Primers used in plasmid construction are listed in Supporting Information (S2 Table). Plasmid constructs in this study were verified by DNA sequencing.

The plasmid pET-28a-*recN* was used for expressing the RecN protein (encoded by *alr4961*). The corresponding fragment was amplified by PCR using primers of Sense-*recN*-pro and Anti-*recN*-pro, with the *Nde*I and *Xho*I sites, and then inserted into the expressing vector pET-28a to give pET-28a-*recN*. The plasmid pRL25T-*recN*-*gfp* was used for expressing the protein translational fusion of *recN* (*recN* in the downstream of its native promoter) and *gfp*_*uv*_. The sequence of the *recN* ORF and its native promoter was amplified by PCR using primer pair Sense-*recN*–983 and Anti-*recN*+1722 with *Not*I and *Pst*I sites, and then inserted into the vector pBS-*gfp*. The fragment obtained with the restriction enzymes (*Not*I and *EcoR*I) was inserted into the shuttle vector pRL25T [34,37], resulting in pRL25T-*recN*-*gfp*.

### Microscopy

Microscopic studies and photographs were carried out using Olympus FV1000 confocal microscope, ZEISS LSM 510 META confocal laser scanning microscope, or Nikon Eclipse 80i microscope (details were provided in related figure legends). DNA was stained by 4, 6-Diamidino-2-phenylindole (DAPI) (Sigma) at 1 mg/mL for 20 min before observation. Images were processed using Olympus Fluoview or Zeiss LSM Image Examiner. 3D analysis was accomplished in Olympus FV10-ASW Version 03.01.01.09. In time-lapse microscopy, filaments were cultured in a chamber slide filled with BG11 medium containing 0.5% agarose and imaged using Nikon Eclipse 80i microscope.

### Western blotting

Western blotting analysis was performed for quantitative determination of RecN protein in vegetative cells and heterocysts. From 100 mL of culture, we collected about 2x10^8^ heterocysts, assuming that about 5% of the cells on the filaments were heterocysts. Total proteins were then prepared from heterocysts samples. Similar amounts of proteins from either heterocyst or vegetative cells were loaded. The amount of protein was determined by Bradford. Antiserum was prepared against purified RecN protein or GFP.

### Electrophoretic mobility shift assay

5-Carboxyfluorescein (5-FAM) labeled dsDNA or ssDNA and RecN were incubated in the binding buffer (50 mM NaCl, 1 mM EDTA, 7.5 mM HEPES, pH 7.5, 1 mM DTT and 20% glycerol) with or without ATP for 30 min at 30°C. Unlabeled dsDNA or ssDNA was added to the mixtures as required. The reaction products were separated in a 5% native PAGE in the running buffer (25 mM tris, pH8.3, 1 mM EDTA, 0.19 M glycine). The labeled complexes were visualized by fluorescence scanner.

## Results

### RecN forms a single dynamic focus in vegetative cells

**Fig 1:**
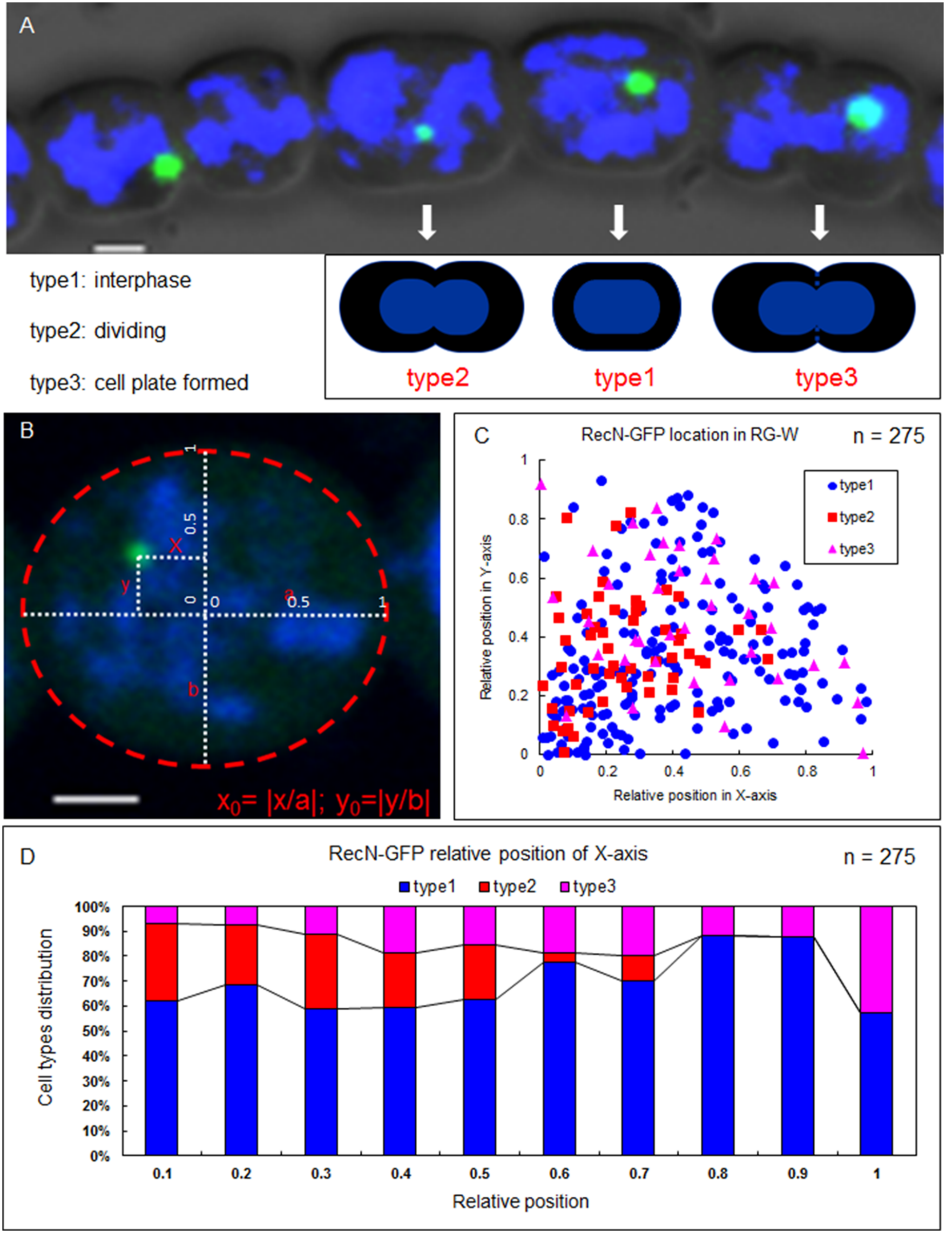
RecN forms a single discrete globular focus in vegetative cells. (A) The three types of vegetative cells divided according to the cell division stages. (B) Localization of RecN-GFP was determined according to its relative position along the x axis and y axis. (C). The locations of the foci in the three types of vegetative cells. The coordinate 0 is the center of the cell. (D) The distributions (in percentage) of the three types of vegetative cells at the relative position of the X-axis. The dynamic localization of RecN-GFP foci was observed with an Olympus FV1000 confocal microscope. Cells were stained with DAPI (blue). Scale bars correspond to 1 μm.

To perform the subcellular localization of the DNA DSB repair protein RecN, we constructed a strain (RG-W) harboring a *recN-gfp* gene fusion with a replicative plasmid in the wild-type *Anabaena*. We compared the expression levels of the wild-type RecN and RecN-GFP by western blotting and determined their relative amounts (S1 Fig). RG-W grows and forms heterocysts like the wild type (data not shown). RecN-GFP displays a single discrete globular focus in most cells (Fig 1A). To better understand the regulation of RecN localization, we divided all cells into three groups according to the division stages. Type 1 cells are at the interphase of cell division; Type 2 cells are divided at the beginning of cell constriction; Type 3 cells are at the end of the division when the cell plate (constricting septum) is formed. We analyzed the relative positions of foci in different cell types as visualized by fluorescence microscopy (Fig 1B). The distribution patterns of Type 1 and Type 3 cells at the relative position of the X-axis and the Y-axis appeared to be random, while Type 2 cells are more likely near the center of the cells (Fig 1C and 1D). Thus, RecN appears to localize as a discrete focus randomly within cells during the interphase of cell division, but it is close to the division center between daughter cells.

**Fig 2:**
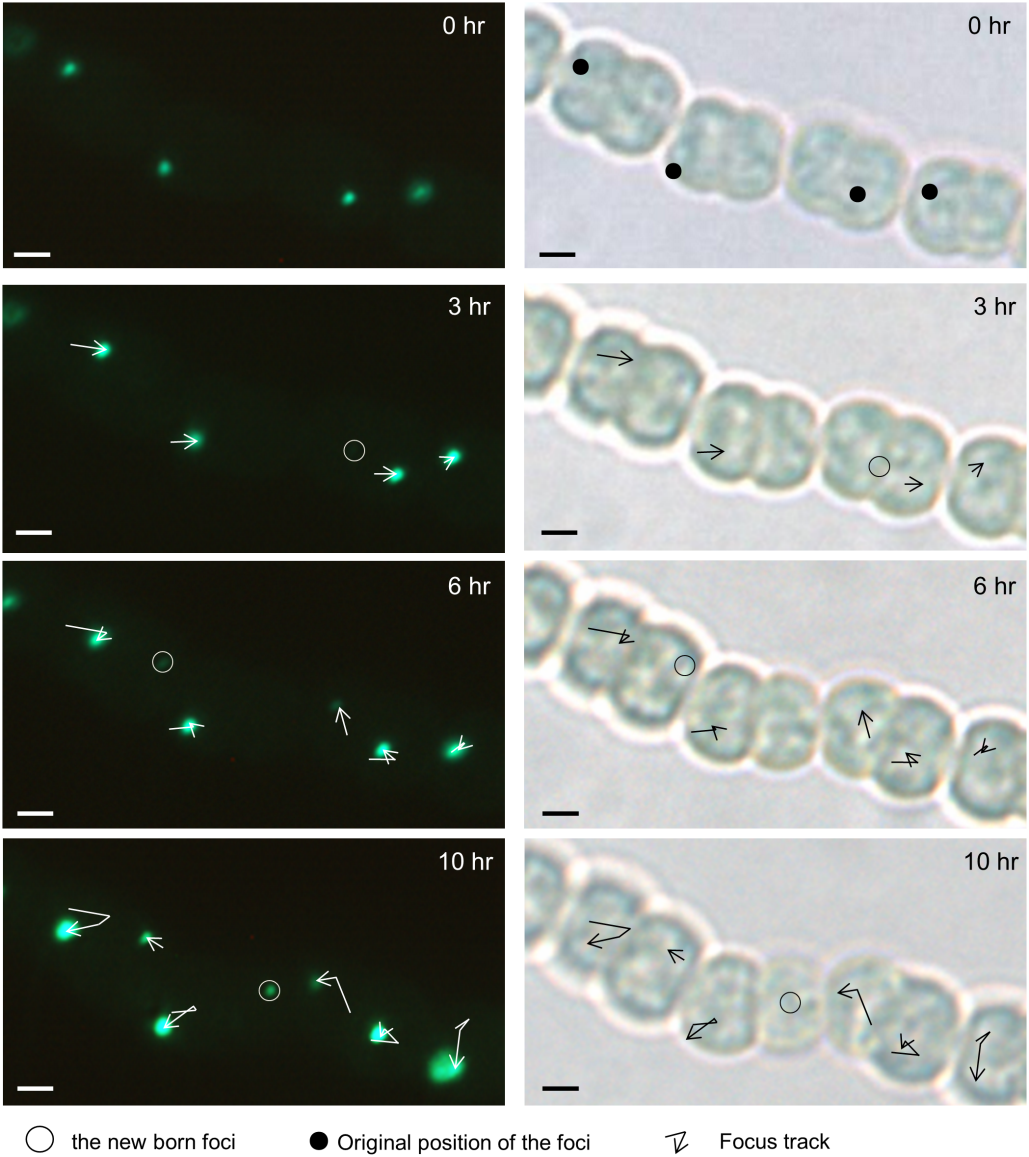
Movement of RecN in vegetative cells displayed by time-lapse imaging. The dynamic localization of RecN focus is demonstrated by time-lapse microscopy from the RG-W. Cells were photographed at 3–4 h intervals. Images on the right were taken in bright field and those on the left were taken in fluorescence respectively in 0, 3, 6, and 10 h. The newborn foci were also marked. Images were taken using a Nikon Eclipse 80i microscope. Scale bars correspond to 1 μm.

To check whether RecN-GFP foci stay at the same position or move around within the cells, vegetative cell filaments were immobilized onto an agarose pad on a slide and photographed every 3–4 h under microscopy. Observation results demonstrated that the foci were dynamically traversing the cells. Single-particle tracking analysis revealed that RecN-GFP foci moved with a random track and that the newborn RecN-GFP foci appeared after the formation of cell septum (Fig 2). In contrast, the movement of the foci was abolished when cells were treated with formaldehyde (S2 Fig).

The RecN-GFP foci were detected both in DNA-occupied and DNA-free areas of the cells. Almost 80% (n = 232) RecN-GFP foci are located in DNA-free areas, while only 20% appeared to be colocalized with nucleoids. However, Z-axis analysis with confocal microscope revealed that some RecN-GFP foci which appeared to be colocalized with DNA in 2-D pictures were actually apart from DNA. Rarely, RecN foci were localized very close to nucleoid, suggesting the possible role of DNA in RecN-GFP dynamics. EMSA analysis showed that *Anabaena* RecN could non-specifically bind to linear ssDNA and that this binding effect was enhanced in the presence of ATP (S3A Fig). RecN could also bind non-specifically to linear dsDNA in an ATP-independent manner (S3B Fig). Similar results were also reported in *Bacillus subtilis*. RecN tethers short ssDNA tails of linear duplex DNA molecules to form a ‘rosette-like’ intermediate structure of homologous recombination in the presence of ATP [11,38]. DNA-binding studies also showed that ATP was not indispensable for DNA binding step of RecN, but it contributed to the formation of RecN protein polymers [7,14].

### Segregation of RecN foci during vegetative cell division

**Fig 3:**
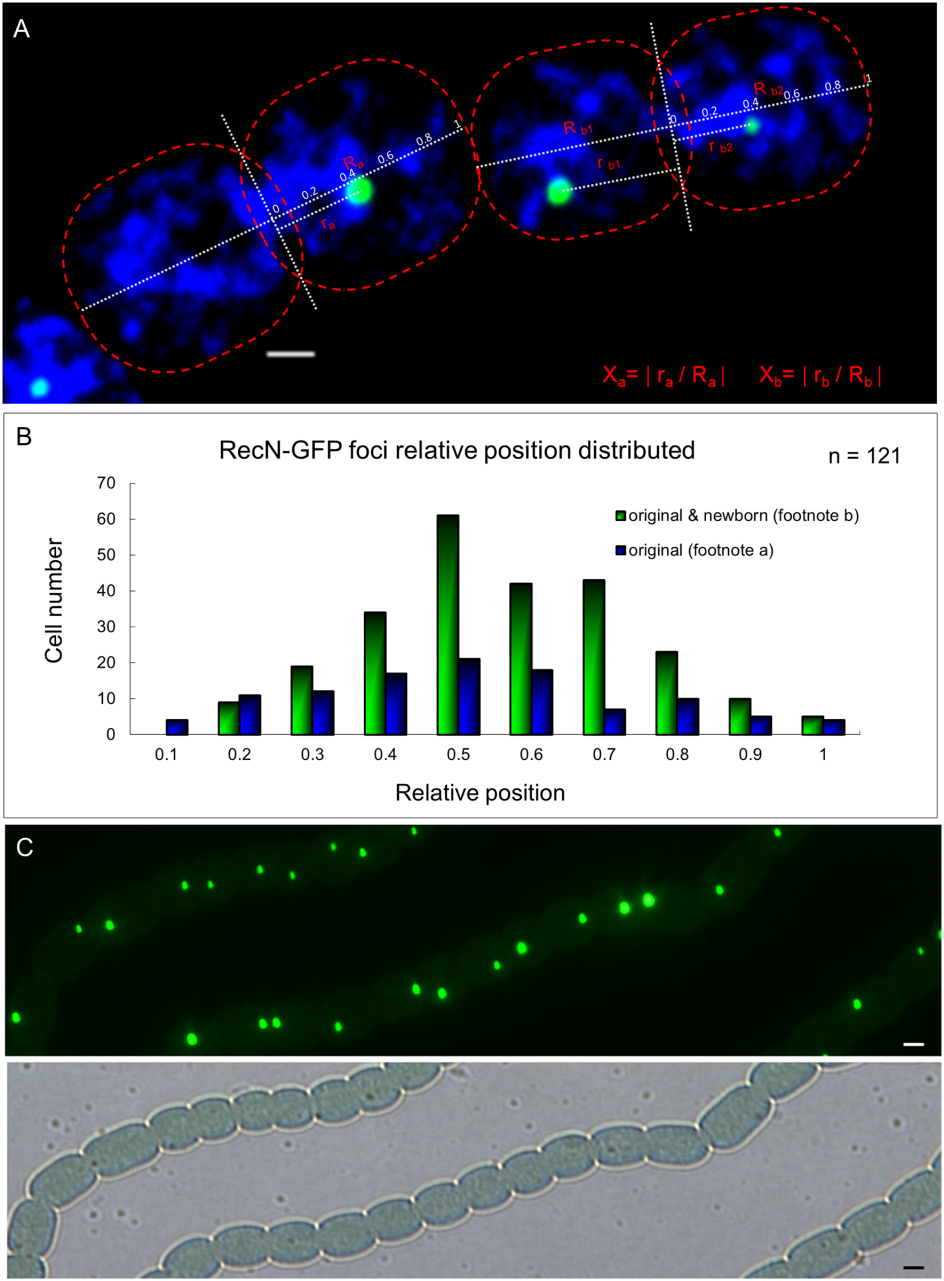
Segregation of RecN foci during vegetative cell division. (A) The method used to quantify the relative distance between foci along the cell division axis. In the formula, “a” means a cell inheriting the original RecN focus after division; “b” means a cell with both an original and a newborn RecN focus. Photographs were captured by using an Olympus FV1000 confocal microscope. Cells were stained with DAPI (blue). Scale bars correspond to 1 μm. (B) Cell number distribution obtained according to the relative distance. (C) Original and newborn RecN foci usually were nearly symmetrically distributed along the cell division axis. Photograph by using a Nikon Eclipse 80i microscope, in the fluorescence and bright fields. Scale bars correspond to 1 μm.

We noticed that some cells did not exhibit a RecN-GFP focus (Fig 1A). These cells corresponded to those which had completed the cell division process and were one of the new daughter cells. Thus, the RecN-GFP focus was partitioned into only one of the two compartments during cell division. Since most cells do possess one RecN-GFP focus, one of the daughter cell should have a newborn focus. To confirm this observation, we analyzed time-lapse images of cells in the late stage of cell division (Fig 2). We found, indeed, that when only one daughter cell inherited the RecN-GFP focus, a new one was produced in the other daughter cell. We further analyzed the relative position of the foci along the cell division axis (Fig 3A). It suggested that newborn and old foci appeared preferentially at the mid-cell position (Fig 3B). When we compared the distance between the two foci along the cell division axis in these dividing cell pairs and found that the two foci usually were nearly symmetrically distributed along the cell division axis (Fig 3C).

### RecN focus moves towards the center of cells under chromosome damaging conditions

**Fig 4:**
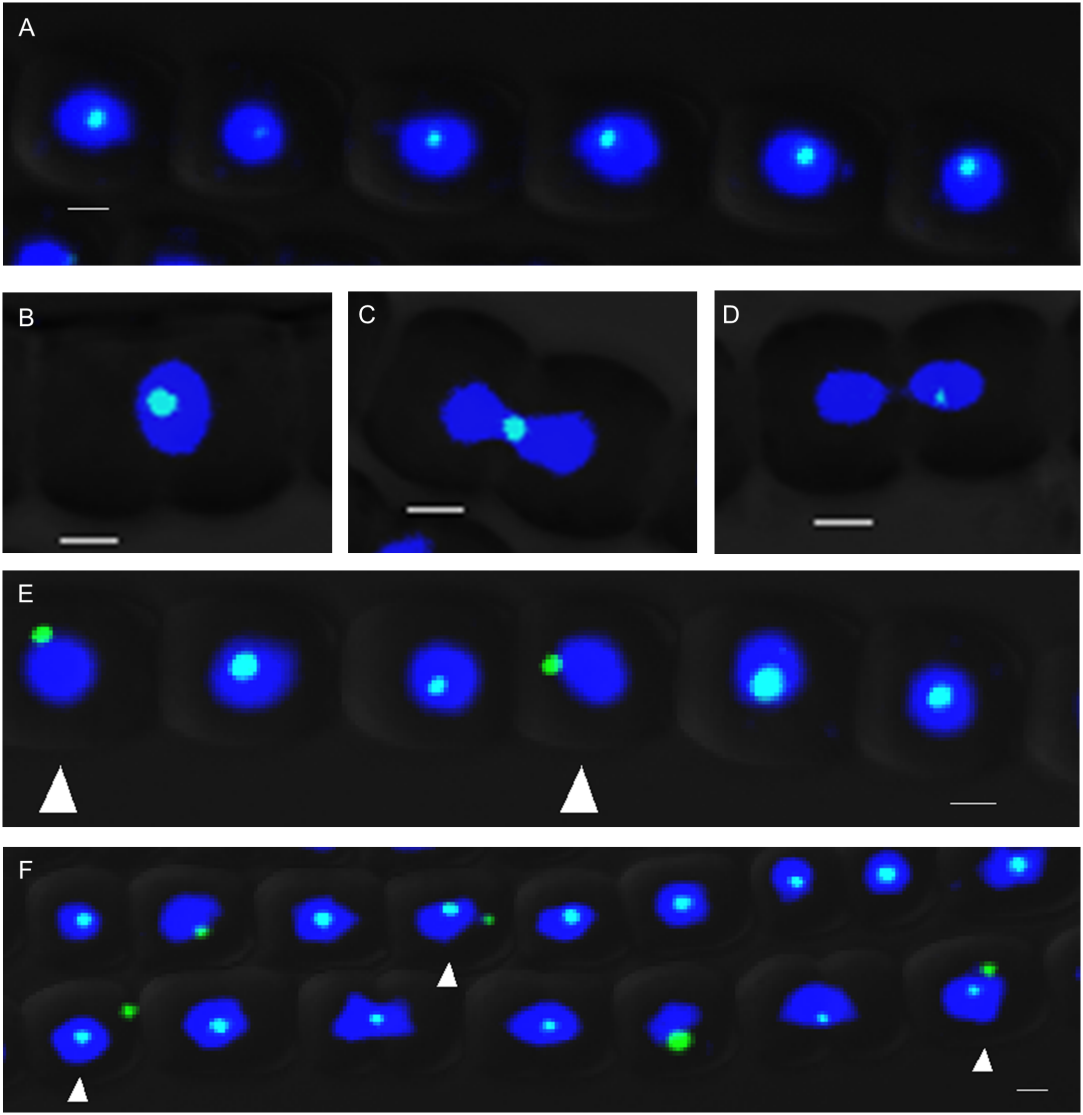
RecN focus is close to the center of the cell treated with MMC. (A) Most RecN-GFP foci are colocalized with nucleoid at the center area of cells when treated with 2 μg/mL MMC. (B, C) RecN-GFP foci and nucleoids were colocalized at the position of cell plate between two daughter cells in dividing cell pairs. (D) RecN-GFP focus was localized in one of the daughter cells when the chromosome was segregated at the later period of cell cycle after MMC treatment. (E) The white arrows indicated that in a few cells, RecN–GFP foci were not colocalized with the chromosome at the central areas (5% in total, n = 196). (F) Nucleoids were more compacted and the number of RecN-GFP foci increased in some cells (white arrows) (21% of cell in a total, n = 213) after the treatment with 4 μg/mL MMC. Photographs were taken by using an Olympus FV1000 confocal microscope. Cells were stained with DAPI (blue). Each photograph involved 3 different channels for DAPI (in blue) GFP (in green) fluorescence and bright field. Scale bars correspond to 1 μm.

If the role of RecN was to repair DSBs, its subcellular localization would be affected by occurrence of DNA lesions. To test and verify this possibility, we determined the subcellular localization of RecN–GFP after the treatment with 2 μg/mL MMC. We found that the majority of the RecN-GFP foci became constantly colocalized with nucleoids at the center of the cells (Fig 4A and S4C Fig). Even in dividing cell pairs, RecN-GFP foci and DNA were colocalized at the position of cell division plane (Fig 4B and 4C). Only in 5% (n = 196) cells, RecN–GFP foci were not colocalized with nucleoids in the central area (Fig 4E), but at the edge of nucleoid. After the chromosome was segregated into two daughter cells, the RecN-GFP focus of the mother cell would be inherited by one of the daughter cells (Fig 4D). The result was similar to that in untreated cells. Furthermore, in 21% (n = 213) of the cells, more than one RecN-GFP focus was found after treatment with MMC at 4 μg/mL (Fig 4F). MMC generates one-ended DSBs related to DNA replication. In contrast, when the cells were treated with nalidixic acid, which inhibited DNA gyrase activity and generated two-ended DSBs, chromosome DNA appeared to be more compacted as a result of the treatment; but few RecN-GFP foci were located at the cell center (S4A and S4C Fig) or at the position of cell division plane in dividing cells(S4B Fig).

### RecN appears as a single discrete globular focus in new heterocysts and is absent in mature heterocysts

**Fig 5:**
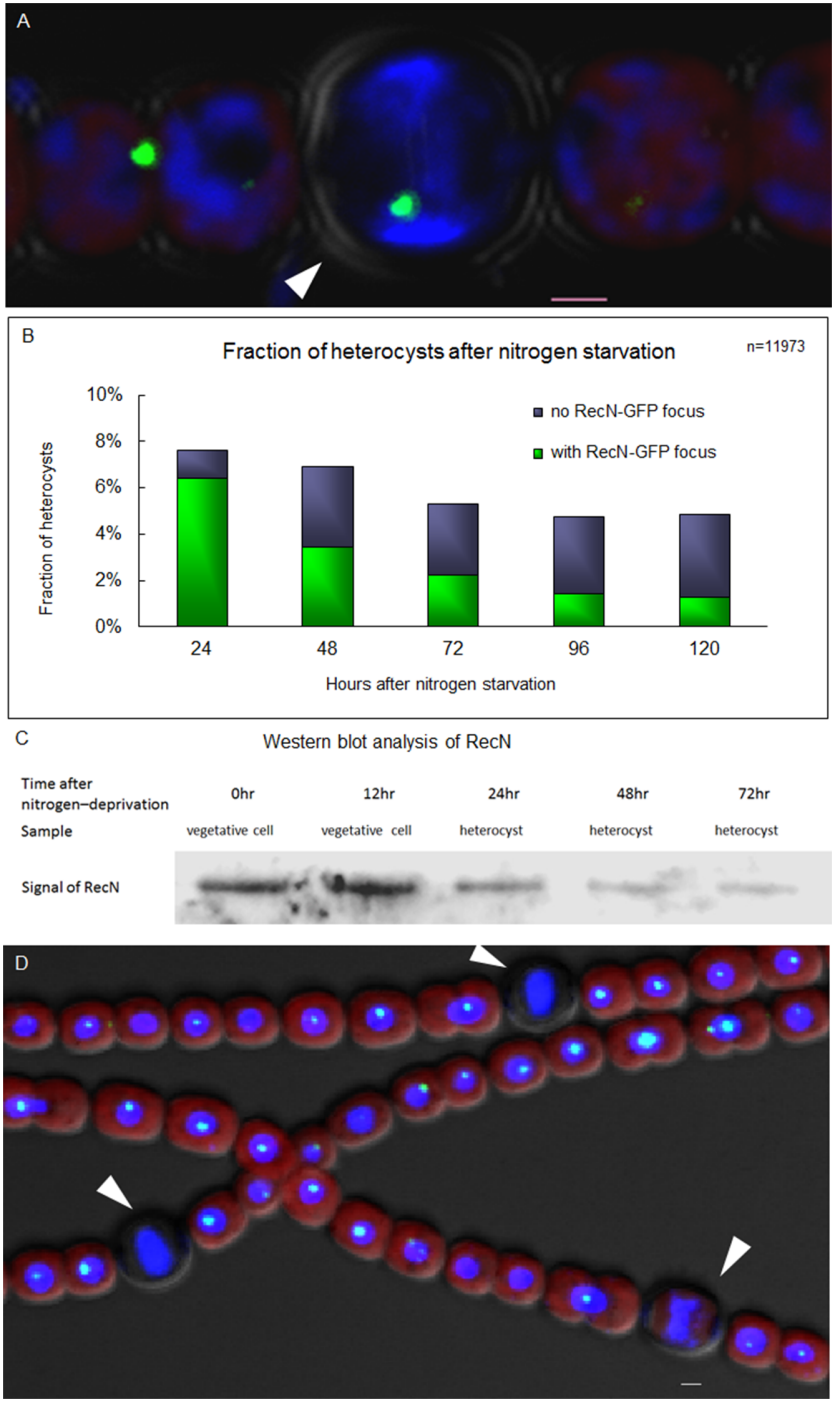
RecN appears as a single discrete globular focus in newly formed heterocyst and disappears in mature ones. (A) RecN-GFP focus in heterocyst (white arrow). (B) Rates (in percentage) of heterocysts with RecN–GFP foci after the starvation of combined nitrogen. (C) Western blotting analysis of protein extracts from vegetative cells or enriched heterocysts using anti-RecN antiserum. Lanes 1 and 2: extracts from vegetative cell in 0 and 12 h after nitrogen deprivation; Lanes 3, 4 and 5: extracts from heterocyst in 24, 48 and 72 h after nitrogen deprivation. (D) High concentration of MMC did not lead to the reformation of RecN-GFP foci in mature heterocysts. After 24 h deprivation of combined nitrogen, 4 μg/mL MMC was added into the medium for 3-day cultivation and then the photographs were captured. The white arrows indicate heterocysts. Photographs were taken by using an Olympus FV1000 confocal microscope. Cells were stained with DAPI (blue). The red fluorescence is the fluorescence of photosynthetic pigment. Scale bars correspond to 1 μm.

Heterocysts are terminally differentiated nitrogen-fixing cells for supplying fixed nitrogen for neighboring vegetative cells. We wonder whether the DNA repair of DSBs is still active in heterocysts because of their terminal nature after differentiation. We performed the subcellular localization of RecN–GFP in heterocysts and found that RecN was localized as a discrete focus in the similar manner to that in vegetative cells (Fig 5A). However, only some heterocysts had a RecN-GFP focus. In a prolonged time after the induction of heterocyst formation, the percentage of heterocysts with a RecN–GFP focus among all heterocysts dropped from 84.2% in 24 h to 26.3% in 120 h (Fig 5B). Consistent with these observations, western blotting also showed that the level of the RecN protein decreased steadily in purified heterocysts after the induction by the deprivation of combined nitrogen (Fig 5C). These observations indicated that a RecN–GFP focus was present in the early stage of heterocyst formation and disappeared overtime in mature heterocysts. Furthermore, 4 μg/mL MMC was added into the medium after 24 h nitrogen starvation, followed by 3-day cultivation. However, most heterocysts still did not display a RecN-GFP focus, in contrast to the results observed in vegetative cells (Fig 5D). DNA appeared more condensed after treatment by MMC in both vegetative cells and heterocysts. These results suggest that the DNA repair system for DSBs may be no longer functional in mature heterocysts.

### RecN localization is affected in a *hetR* mutant

**Fig 6:**
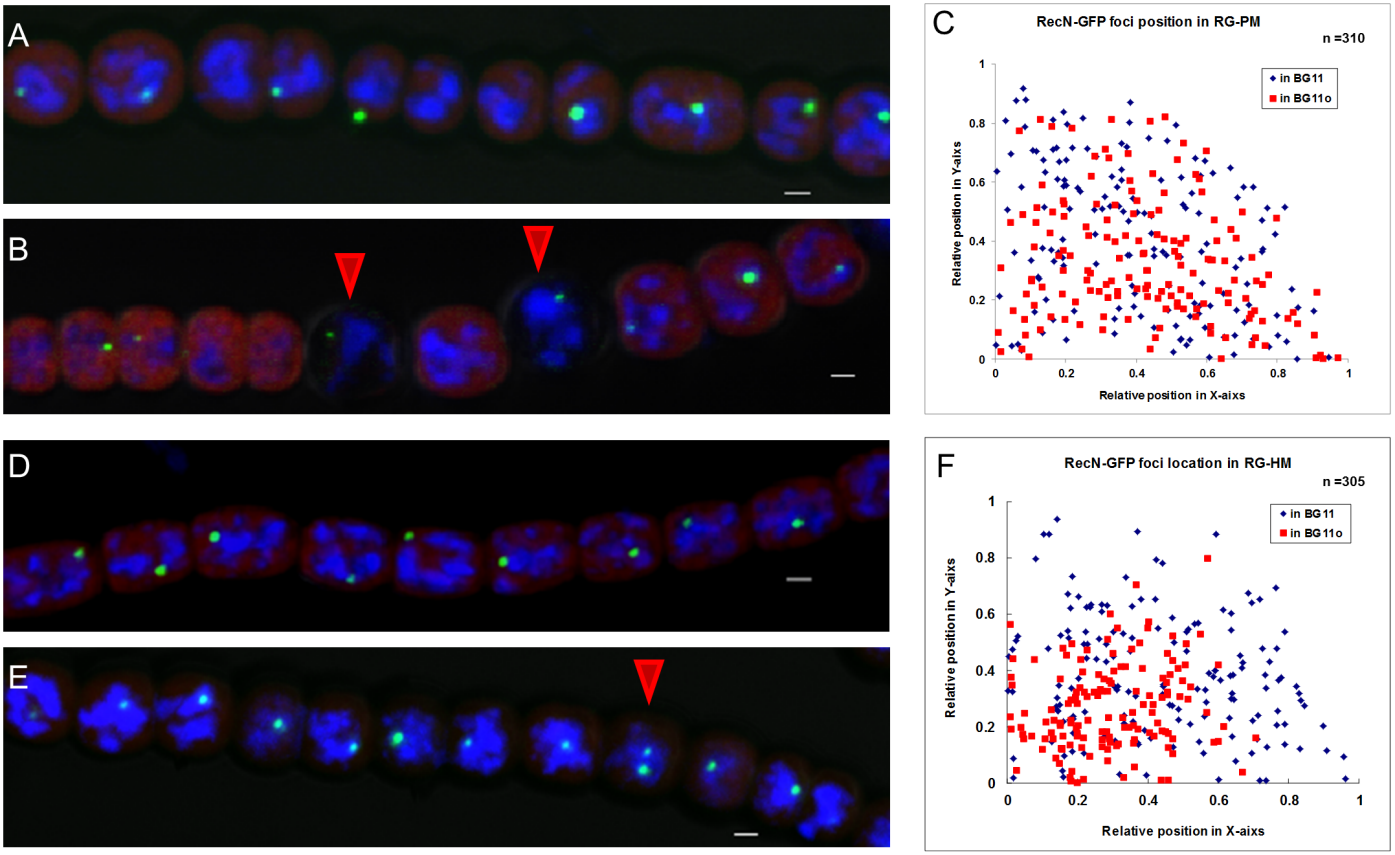
RecN localization is affected in patS or hetR mutant. (A) RecN-GFP foci in the strain RG-PM cultured in the medium BG11. (B) RecN-GFP foci in the strain RG-PM cultured in the medium BG11_0_. The red arrows indicate heterocysts. (C) The localization of RecN-GFP foci in the strain RG-PM cultured in different media. The coordinate 0 is the center of the cell. The statistical method used here was the same with that in Fig 1B. (D) RecN-GFP foci in the strain RG-HM cultured in the medium BG11. (E) RecN-GFP foci in RG-HM cultured in the medium BG11_0_, the red arrow indicates a cell with 2 foci. (F) The localization of RecN-GFP foci in the strain RG-HM cultured in different media. The coordinate 0 is the center of the cell. The statistical method used here was the same with that in Fig 1B. Photographs were taken by using an Olympus FV1000 confocal microscope. Cells were stained with DAPI (blue). The red fluorescence is from the photosynthetic pigments. Scale bars correspond to 1 μm.

Our data showed that a RecN focus was present at the early stage of heterocyst differentiation, but disappeared in most mature heterocysts. We further performed RecN-GFP localization in a *patS* mutant UHM114 [39], where the *patS* gene was deleted, and a *hetR* mutant hetR216 carrying a loss-of-function point mutation [40,41]. The *patS* gene encodes an inhibitor of heterocyst differentiation, while *hetR* encodes a transcription factor required for heterocyst development [24,25]. Two genes, *patS* and *hetR*, are involved in heterocyst differentiation and patterning in *Anabaena* [42,43]. Under nitrogen deprivation conditions, UHM114 develops into heterocysts at a higher frequency as compared to the wild type strain of *Anabaena*, whereas hetR216 has no capability to form heterocysts [25]. The UHM114 strain with expressed RecN-GFP is named as RG-PM, while the hetR216 strain with expressed RecN-GFP is named as RG-HM. When cultured in BG11 medium containing a combined nitrogen, both RG-PM and RG-HM showed a similar location of RecN-GFP foci to that observed in RG-W (wild-type contrast), displaying a single discrete focus at DNA-free parts of the cells (Fig 6A and 6D). In the nitrogen–deprivation medium BG11_0_, RG-PM developed more heterocysts as expected, but the localization of RecN-GFP foci was not different from that in RG-W (Fig 6B and 6C). However, the RG-HM strain showed two differences from RG-W under the same culture conditions. First, RecN-GFP foci in RG-HM cells were mostly colocalized with nucleoid-occupied regions at the center area of cells (Fig 6E and 6F). Secondly, 12.3% of the cells had 2 or 3 foci (Fig 6E). The phenomenon was observed in RG-W cells only after treatment by Mitomycin C.

## Discussion

RecN was found to play a role in the RecF-dependent gap repair pathway and was involved in the RecBCD DSB repair pathway [13]. RecN is also required for the suppression of chromosomal rearrangements and deletions [17]. In *Bacillus subtilis*, after one-ended and two-ended DSBs, RecN foci are colocalized with the damage site and close to the ‘replication factory’ [19,21]. DNA replication is part of recombinational repair of DNA damage process, especially for one-ended DSBs. Therefore, it is probable that RecN and its partners together with damaged DNA are located at the center of the cells to facilitate DNA repairing with the aid of DNA replication machinery when DNA damage is particularly strong. In *Anabaena*, RecN is colocalized to DNA-occupied areas of the cells at the time of DNA lesions. When the cells were treated with MMC, the number of foci in some cells increased, mostly at the center of the cells or at the connection of two daughter cells (Fig 4B and 4C). DnaA, a protein for DNA replication initiation, is located at the center of the cells in *Anabaena* (S5 Fig), suggesting a possible relationship between DNA repair and DNA replication in this organism. The phenomenon is similar to that found in *Bacillus subtilis*. Paula P. Cardenas proposed that RecN, perhaps in association with PNPase, was one of the first responders to DNA DSBs, serving as a ‘sentinels’ for checking DNA integrity [20]. The dynamic movement of RecN in *Anabaena* reported here is consistent with this proposed role. Unfortunately, we were unable to obtain a mutant of *recN*, preventing us at this stage from a better understanding of the role of RecN in *Anabaena*.

We found a different pattern of RecN localization in the two types of cells, namely, heterocysts and vegetative cells. RecN appears to be present at early stages of heterocyst differentiation, but disappears in mature heterocysts. MMC treatment does not lead to the reformation of RecN foci in these heterocysts. This finding raises the question whether DSBs repair of DNA is still operational in heterocysts. Heterocysts are terminally differentiated cells and unable to be divided [24,27,44], but they can still supply fixed nitrogen to vegetative cells. Furthermore, Nürnberg et al. reported that some heterocysts might lose the ability of metabolic communication with vegetative cells in 48 h [45], suggesting that some heterocysts might lose the ability to provide fixed nitrogen to vegetative cells. Thus, DNA fidelity or the survival of heterocysts may not be as critical as that in vegetative cells. If DNA damages are accumulated to a level at which a heterocyst becomes nonfunctional, a new one is formed to support the nitrogen supply of vegetative cells.

The known functions ascribed to HetR were related to heterocyst development, but we found a new phenotype associated with *hetR* mutation (Fig 6E). Under the deprivation of combined nitrogen, vegetative cells of *hetR216* allele display a pattern of RecN-GFP that is similar to those treated by MMC in the wild type. Whether *hetR* is involved in the maintenance of DNA integrity during heterocyst development is of great significance for further investigation.

## Supporting Information

S1 Fig Western blotting analysis of RecN and GFP in wild and RG-W strains. Western blotting analysis of protein extracts from the wild-type strain and RG-W using anti-RecN serum (left) or anti-GFP serum (right). Strain name are marked at the top of each lane.
(TIF)

S2 Fig Movement of RecN in vegetative cells displayed by time-lapse imaging after the treatment with formaldehyde. The localization of RecN foci in vegetative cells after the treatment with 1% formaldehyde followed by time-lapse microscopy from the RG-W. Cells were photographed at 3–4 h intervals. Images on the right were taken in the bright field and those on the left were taken in fluorescence in 0, 3, 6, and 10 h. The positions of foci were also marked in the bright field. Images were taken using a Nikon Eclipse 80i microscope, scale bars correspond to 1 μm.
(TIF)

S3 Fig DNA-binding activity analysis of *Anabaena* RecN. ssDNA-binding activity Analysis of RecN. The reactions contained 0.3 μM 5-FAM labeled ssDNA_1_ (Lanes 1–8); 0.3, 0.6, and 1.0 μM ssDNA_2_ (Lanes 6–8) or no ssDNA_2_ (Lanes 1–5); 0, 0.008, 0.02 (Lanes 1–3) and 0.04 nM RecN (Lanes 4–8); ATP (1 mM) was only present at Lane 5 (Figure A). Analysis of dsDNA-binding activity of RecN. Reactions contained 55 μM 5-FAM labeled dsDNA_1_ (Lanes 1–7); 10, 50, and 250 nM dsDNA_2_ (Lanes 5–7) or no dsDNA_2_ (Lanes 1–4); 0, 530, 1325 (Lanes 1–3) and 2650 ng RecN (Lanes 4–7) (Figure B). 5-FAM radical group was indicated by asterisk; P·D, Protein–DNA complexes; FD, free DNA. The sequences of ssDNA and dsDNA were also listed in Supporting Information (S1 File).
(TIF)

S4 Fig Subcellular localization of RecN after treatment with nalidixic acid. Subcellular localization of RecN in vegetative cells treated with 500 μg/mL nalidixic acid (Figure A). Subcellular localization of RecN in dividing cell pairs treated with 500 μg/mL nalidixic acid (Figure B). The localization of RecN-GFP foci location under the treatment by MMC or nalidixic acid. The coordinate 0 is the center of the cell. The statistical method used here was the same with that in Fig 1B (Figure C). Photographs were taken by Olympus FV1000 confocal Microscope. Cells were stained with DAPI (blue). Scale bars correspond to 1 μm.
(TIF)

S5 Fig DnaA-GFP foci in *Anabaena* PCC7120. Subcellular localization of DnaA-GFP in filaments of *Anabaena* (strain DG-HM) (Figure A). Subcellular localization of DnaA-GFP in dividing cell pairs (Figure B-D). The localization of DnaA-GFP foci in *Anabaena*. The coordinate 0 is the center of the cell. The statistical method used here was the same with that in Fig 1B (Figure E). Coordinate origin is the center of the cell. Photographs were taken by ZEISS LSM 510 META confocal laser scanning microscope. Scale bars correspond to 2 μm.
(TIF)

S1 File Sequences for EMSA Analysis. (DOC)

S1 Table Strains and plasmids used in this study. (DOC)

S2 Table Primers used in this study. (DOC)
