# Supplementary figures and images for: Dynamics and Cell-Type Specificity of the DNA Double-Strand Break Repair Protein RecN in the Developmental Cyanobacterium Anabaena sp. Strain PCC 7120
Source: PLoS One. 2015 Oct 2;10(10):e0139362. doi: 10.1371/journal.pone.0139362 (PMC4592062; doi:10.1371/journal.pone.0139362)

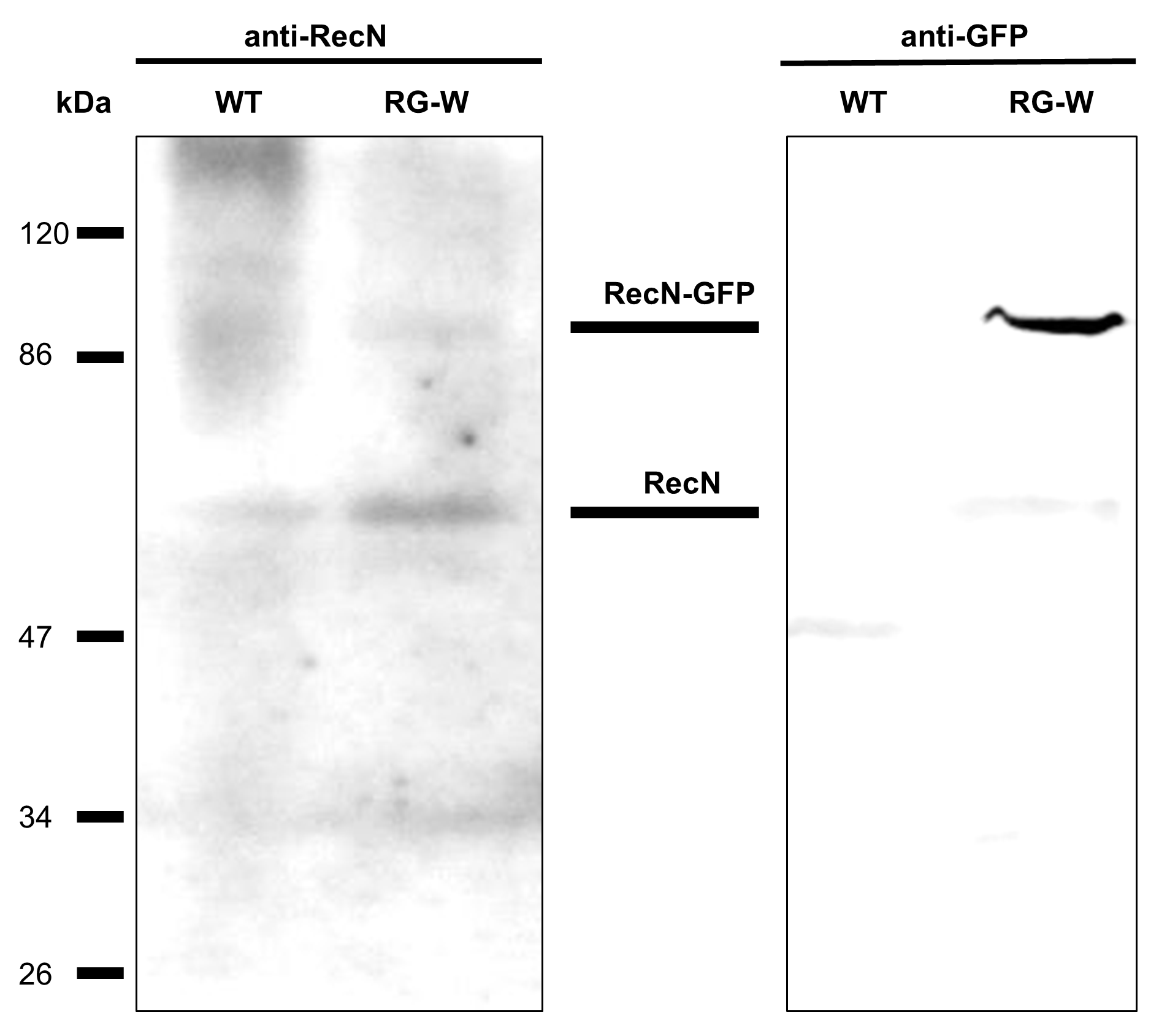

Supplement: S1 Fig — Western blotting analysis of protein extracts from the wild-type strain and RG-W using anti-RecN serum (left) or anti-GFP serum (right). Strain name are marked at the top of each lane. (TIF) [file pone.0139362.s001.tif]

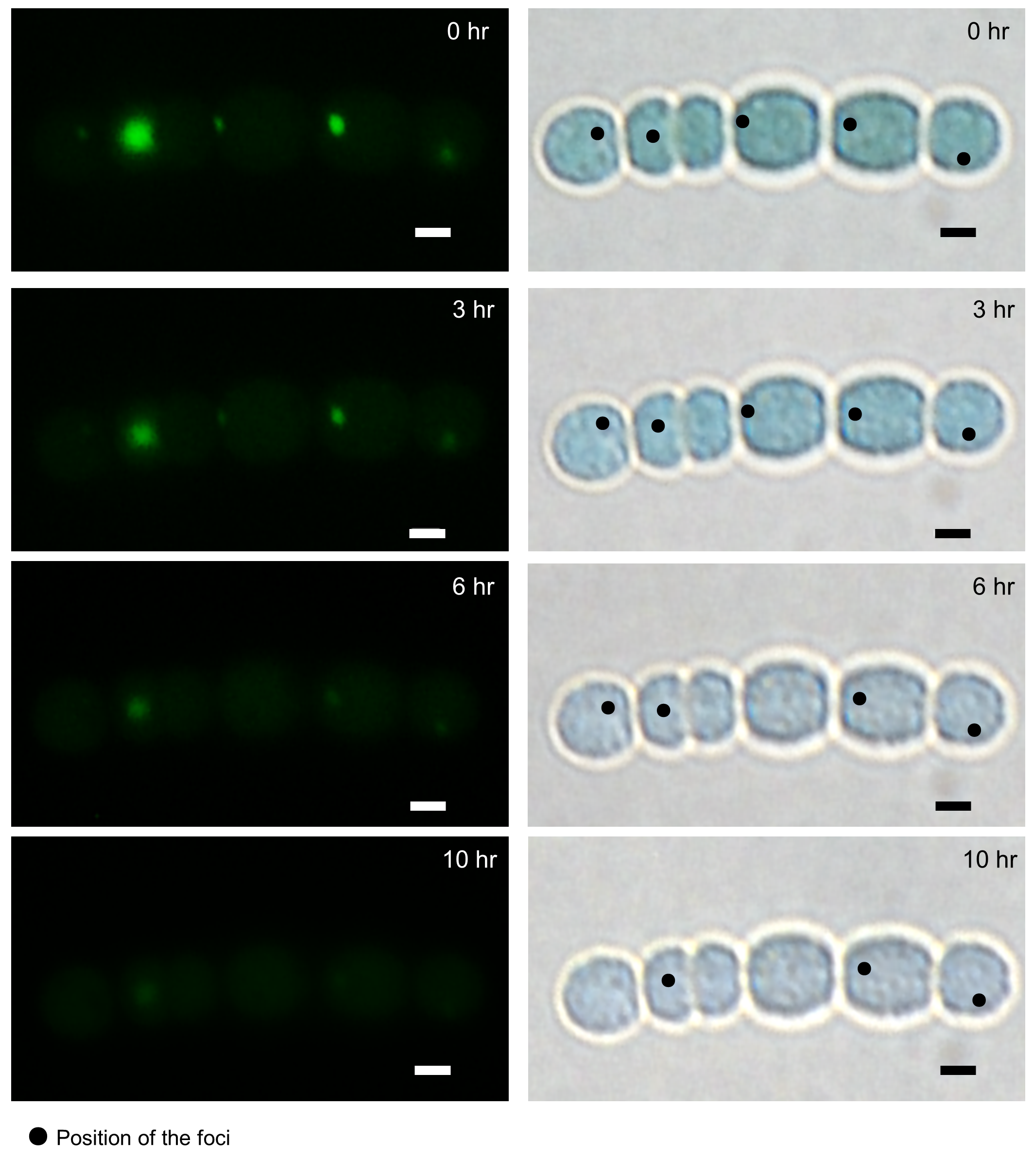

Supplement: S2 Fig — The localization of RecN foci in vegetative cells after the treatment with 1% formaldehyde followed by time-lapse microscopy from the RG-W. Cells were photographed at 3–4 h intervals. Images on the right were taken in the bright field and those on the left were taken in fluorescence in 0, 3, 6, and 10 h. The positions of foci were also marked in the bright field. Images were taken using a Nikon Eclipse 80i microscope, scale bars correspond to 1 μm. (TIF) [file pone.0139362.s002.tif]

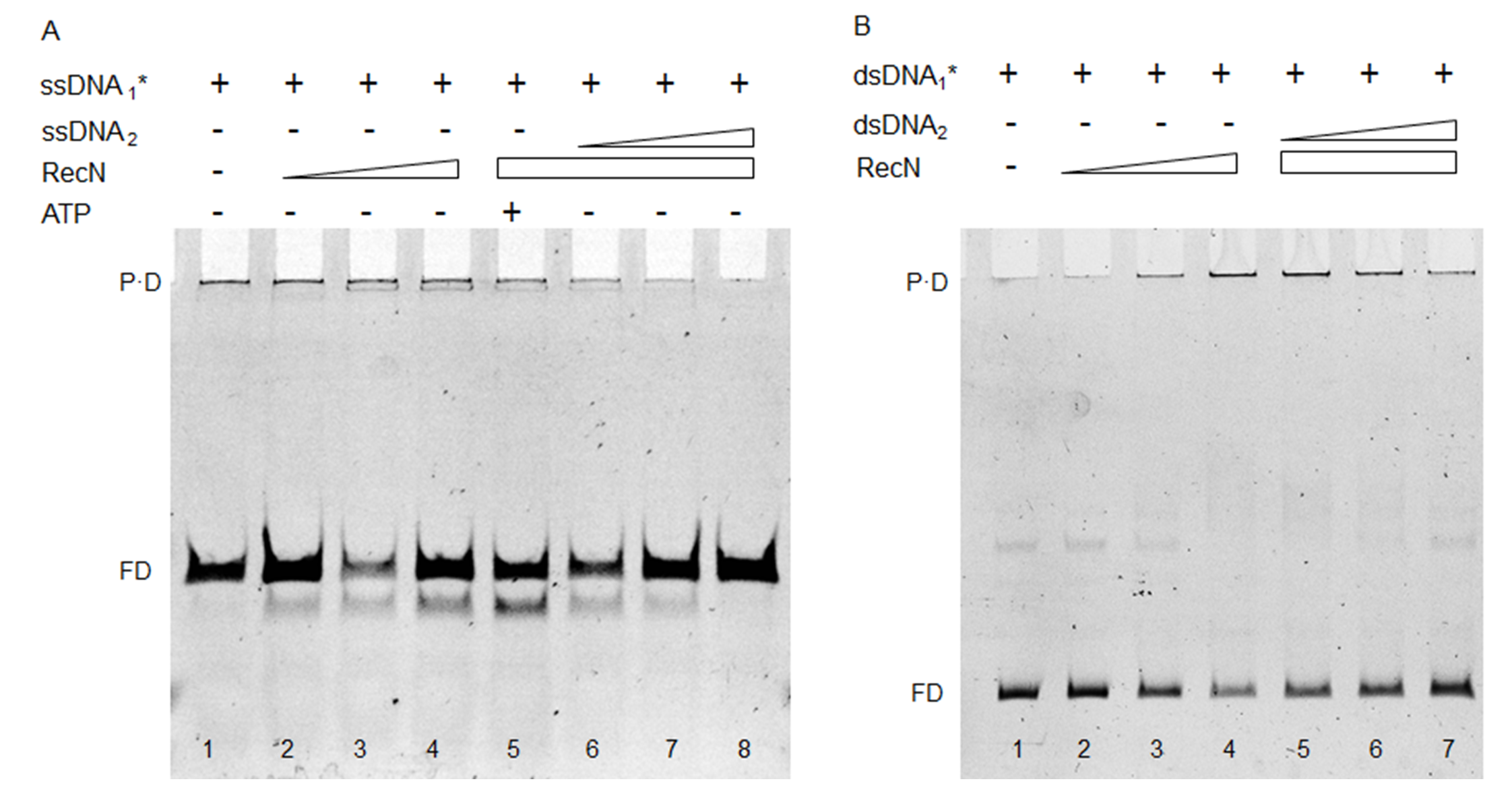

Supplement: S3 Fig — ssDNA-binding activity Analysis of RecN. The reactions contained 0.3 μM 5-FAM labeled ssDNA1 (Lanes 1–8); 0.3, 0.6, and 1.0 μM ssDNA2 (Lanes 6–8) or no ssDNA2 (Lanes 1–5); 0, 0.008, 0.02 (Lanes 1–3) and 0.04 nM RecN (Lanes 4–8); ATP (1 mM) was only present at Lane 5 (Figure A). Analysis of dsDNA-binding activity of RecN. Reactions contained 55 μM 5-FAM labeled dsDNA1 (Lanes 1–7); 10, 50, and 250 nM dsDNA2 (Lanes 5–7) or no dsDNA2 (Lanes 1–4); 0, 530, 1325 (Lanes 1–3) and 2650 ng RecN (Lanes 4–7) (Figure B). 5-FAM radical group was indicated by asterisk; P·D, Protein–DNA complexes; FD, free DNA. The sequences of ssDNA and dsDNA were also listed in Supporting Information (S1 File). (TIF) [file pone.0139362.s003.tif]

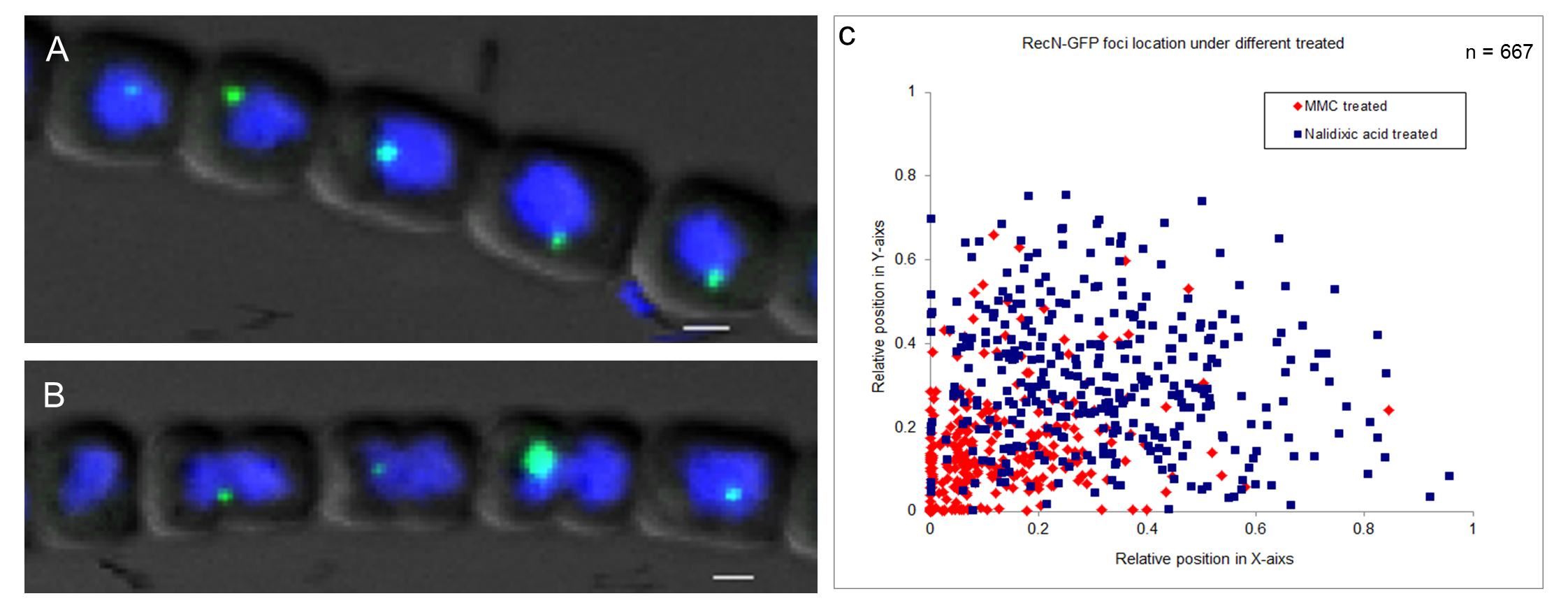

Supplement: S4 Fig — Subcellular localization of RecN in vegetative cells treated with 500 μg/mL nalidixic acid (Figure A). Subcellular localization of RecN in dividing cell pairs treated with 500 μg/mL nalidixic acid (Figure B). The localization of RecN-GFP foci location under the treatment by MMC or nalidixic acid. The coordinate 0 is the center of the cell. The statistical method used here was the same with that in Fig 1B (Figure C). Photographs were taken by Olympus FV1000 confocal Microscope. Cells were stained with DAPI (blue). Scale bars correspond to 1 μm. (TIF) [file pone.0139362.s004.tif]

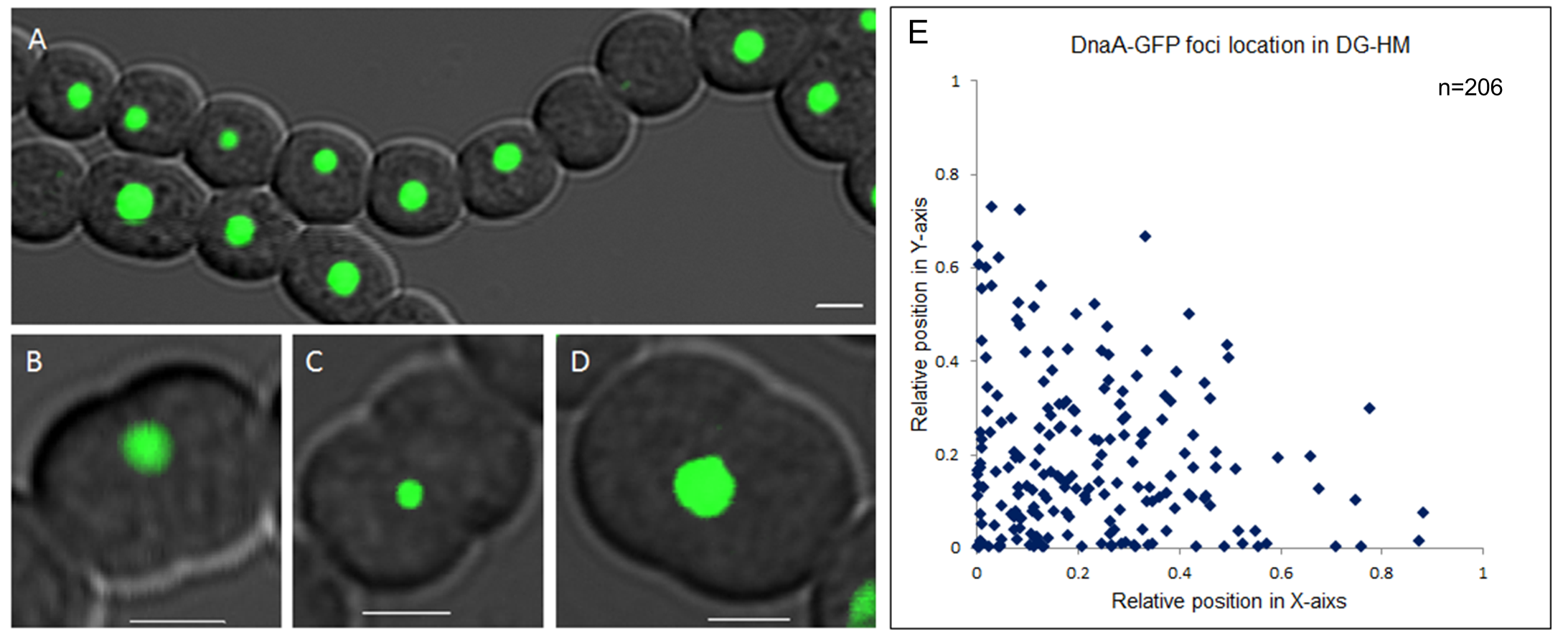

Supplement: S5 Fig — Subcellular localization of DnaA-GFP in filaments of Anabaena (strain DG-HM) (Figure A). Subcellular localization of DnaA-GFP in dividing cell pairs (Figure B-D). The localization of DnaA-GFP foci in Anabaena. The coordinate 0 is the center of the cell. The statistical method used here was the same with that in Fig 1B (Figure E). Coordinate origin is the center of the cell. Photographs were taken by ZEISS LSM 510 META confocal laser scanning microscope. Scale bars correspond to 2 μm. (TIF) [file pone.0139362.s005.tif]
