# Supplementary material for: Dynamics and Cell-Type Specificity of the DNA Double-Strand Break Repair Protein RecN in the Developmental Cyanobacterium Anabaena sp. Strain PCC 7120
Source: PLoS One. 2015 Oct 2;10(10):e0139362. doi: 10.1371/journal.pone.0139362 (PMC4592062; doi:10.1371/journal.pone.0139362)
Supplement: S1 File — (DOC) [file pone.0139362.s006.doc]

**S1 File. Sequences for EMSA Analysis**

**ssDNA_1_:**

AAAGGAGGTGATCCAGCCACA

**ssDNA_2_:**

CCTTCATATGGCTTTTCGACCCCGCAAACGAT

**dsDNA_1_:**

TGTCAAGCTACCTGCTGACTCTACATCATAAATCTAGTGCAAATTTATATCCATATACTATTGGTAAGCAACCATTAGCATTTATATATGAATGTGAAAAATTCACTGAAATTTGATCAGCTAATTTTTCACGTTAAATAATTGCTTATCAACCTATTTAGTGGTAAATTCTCTTAGTAGGTTTAAAGTCTCAAATGCACAGTAGGCGAGGGGTCTAACCCCTCATTACCTAGTAGAAAATGCTCAAAATCGTCAGCAGGTAGCTTGACA

**dsDNA_2_:**

GAATGCGGCCGCGGGAGTTCCTGGGTTAGGGTCAATTACTCGACTCCATGAAGCCAAACCATCAGCAATACCCAAAGCGAAATCGCGCCGACGATTTTGCAGCAAAGCCCGATCCTCAGGACTGCTGAGAAAAGCCACCTGCATGAGTAAAGAAGCGATCGCAATCTGCCGACAAAAGGCTAATCTACCCAAACCACTGTCTGTATCTGGTTTAACACCCCGATTTGGTAACTGGGGTACACGGCGTAACAATCCAACTAACAGCAATTCCCCGTTGCTTTTACGCTCATTATTACTGGCAATATAAAACACACTAGCACCCCGCACAGCCGGACTACTCGCCGAATCAGCATGGACTTCTAGGGATACATCACCTCGACGACCACGAGCATTGATCCAGGCGATAGTATCAGCCGCACTCAAATCATCGGGAACAGACAAAACTTCAAAACTCCGCGATCGCAATTCTGTCACAATTAAATCTCGTAACAGAATCATCTCTCTAGCTTCCGTCGTTCCCCCCGCGATTGAGCCTGGATCTACTCCTCCCGCTTCTCTGCCTCCGTGGGCTGCCGAAATAAAAATACGCCCCATCTCCCGTATTTCCTCTGATAAAATTAAGCGTAAGCAATTCTATACAATATTTTTGGCTTGGCAACAATCATTATATGGGGAGTGGGAATAGATATACTGACCACTGAACACTGACAACTGTACAGACGCGATTAATCGCGTCTCTGACAACTGACAAGTAACAAAAATGCAAATTCCGCGCCTCCATCCAGATACGATTGATGAAGTGAAACATCGGGCTGATATTGTCGATGTGGTATCGGAATATGTGGTTTTACGCAAGCGGGGTAAAGATTTTGTCGGCTTGTGTCCTTTCCATGATGAAAAATCTCCTAGTTTTACTGTCAGCCAAACTAAGCAAATGTATTATTGCTTCGGTTGTCAGGCTGGGGGAAATTCGATTAAGTTTTTGATGGAATTGGGCAAGCGATCGTTTGCGGATGTGGTGTTAGATTTAGCTAGGCGCTACCAAGTCCCAGTCCAAACCCTGGAACCAGAGCAAAGGCAAGAATTACAGCGTCAGATATCGTTGCGGGAACAGTTGTATGAGGTGCTGGCTTCGGCGGCTAGTTTTTATCAACACGCCCTCAGACAATCACAAGGACAAAGGGCGATGCAGTATTTACTAAGCGATCGCCAATTTAATGAAGTAACAATCCAACAGTTCGGCTTGGGTTATGCGCCCGCAGGTTGGGAAACTCTCCATCGTTATTTGGTGGAAGATAAACATTATCCAGTGCATCTGGTAGAAAAAGCAGGTTTGATTAAACCGCGTAAGGAAGGCGCTGGATATTATGATGTGTTCCGCGATCGCCTCATGATTCCCATCCGCGATATCCAAGGACGGGTAATTGGCTTTGGTGGACGAACTTTAACGGATGAACAACCGAAGTATTTAAATTCACCAGAAACCGAACTATTTAATAAAGGTAAAACCCTCTTCGCCCTCGACCAAGCCAAAGCGGGGATTTCCCAGTTTGATCAAGCCGTGGTGGTGGAAGGTTATTTTGATGCGATCGCTCTCCACGCAGCCGGAATTAATCATGCTGTGGCTTCTTTAGGTACTGCCCTGAGTTTAGAACAAGTCCGGCTAATATTACGCTACACCGACTCAAAACAGTTGGTACTCAACTTTGATGCAGATAAAGCCGGAACCAACGCCGCCGAAAGGGCGATCGGGGAAATTGCCGATTTAGCTTACAAAGGCGAAGTGCAACTGAAAATTCTCAATATCCCCGATGGCAAAGATGCTGATGAATATCTGCGTAGCCATACACCAGCAGATTACGGGCAATTATTAGCAAATGCGCCCTTATGGTTGAACTGGCAGATTCAGCAGATTATCAAAGACCGCGATTTAAAACAAGCGACTGATTTTCAGCAAGTTACACAGCAATTTGTCAAACTACTCAAAAATATAGTTAATAGTGATACCCGTAACTATTATGTTTCTTACTGCGCGGAAATCCTCAGCTTAGGAGACACCAGACTCATCCCCCTGCGAGTGGAAAATCTCCTCACGCAAATCACCCCAACTACCGCCGGCTATAACAAACCCCTGTCAACCAGGACGAATAGAAGCCTTTCCCTAGTCCCCAACCCTACTTCTTCACTCAATGGCGATCGCAGCCTGCTAGAACTAGCTGAGGCGCTACTACTGCGGATTTACCTCCATTGTCCCGAACAGCGTCGAGTGATTATGGAAGAACTAGAAGCCAGAAATTTAGAGTTTAGCCTATCCCACCATAGATTTTTGTGGCAACAGATTTTAGAATGCACAGGTGAACAGGTTGATTTAGTTTCCCGATTGCAAGATATATATTTAGAAATAGCTGAAGAACTTGGGGTAATTTCTCATCTGTTTCATCTCAATGAGAAAACCAAGAAAGAGATCATGCGGACTACCCAAGTTGTACAAGCAGCGATCGCCTGTATGGAACGAGTGTTAACAGAAAAGCGTTATCGTCACTTTCTGGAACTGTGGCAAGAAACTGATCCTGAAACTGAACCAGAAAAATGGCAATCATATTATCAGGCATTCTACACCGAAAAAATCAAGCTACAAGAACTAGACCGACAACGCCAATTTTCCCTAACAGAATTACTATAACTGCAG TTGG
