# Supplementary material for: Dynamics and Cell-Type Specificity of the DNA Double-Strand Break Repair Protein RecN in the Developmental Cyanobacterium Anabaena sp. Strain PCC 7120
Source: PLoS One. 2015 Oct 2;10(10):e0139362. doi: 10.1371/journal.pone.0139362 (PMC4592062; doi:10.1371/journal.pone.0139362)
Supplement: S1 Table — (DOC) [file pone.0139362.s007.doc]

**S1 Table. Strains and plasmids used in this study.**

| **Strain/plasmid** | **Description** | **Source** |
| --- | --- | --- |
| ***Cyanobacteria* strains** | | |
| ***Anabaena* sp. PCC 7120** | Wild type (WT) | Pasteur Culture Collection |
| ***hetR216*** | point mutation in the *hetR* |  |
| **UHM114** | Δ*patS* |  |
| **RG-W** | Neo^r^, pRL25T-*recN-gfp* introduced into WT strain | This study |
| **RG-HM** | Neo^r^, pRL25T-*recN-gfp* introduced into *hetR216* strain | This study |
| **RG-PM** | Neo^r^, pRL25T-*recN-gfp* introduced into UHM114 strain | This study |
| **DG-HM** | Neo^r^, pRL25T-*dnaA-gfp* introduced into *hetR216* strain | This study |
| ***E. coli* strains** | | |
| **DH5α** | F^-^, *supE*44 *Δ(lacZYA-argF) U*169 (Φ80*lacZΔM*15) *hsdR*17 *recA endA*1 *gyrA*96 *thi*-1 *relA* 1 | ATCC |
| **BL21(DE3)** | F^-^, *ompT gal dcm lon hsdS_B_(r_B_^-^ m_B_^-^)* λ(DE3 *[lacI lacUV5-T7 gene 1 ind1 sam7 nin5])* | Novagen,USA |
| **Plasmids** | | |
| **pET-28a-c(+)** | Expressing vector | Novagen,USA |
| **pET-28a-*recN*** | pET-28a carrying *recN_ana_* ORF | This study |
| **pRL25T** | Km^r^ Nm^r^, pDU1-based shuttle vector | . |
| **pRL25T-*recN-gfp*** | Km^r^ Nm^r^, pRL25T containing *recN_ana_* ORF with native promoter, and *gfp* coding sequence | This study |
| **pRL25T-*dnaA-gfp*** | Km^r^ Nm^r^, pRL25T containing *dnaA_ana_* ORF with native promoter, and *gfp* coding sequence | This study |
