# Supplementary material for: Dynamics and Cell-Type Specificity of the DNA Double-Strand Break Repair Protein RecN in the Developmental Cyanobacterium Anabaena sp. Strain PCC 7120
Source: PLoS One. 2015 Oct 2;10(10):e0139362. doi: 10.1371/journal.pone.0139362 (PMC4592062; doi:10.1371/journal.pone.0139362)
Supplement: S2 Table — (DOC) [file pone.0139362.s008.doc]

**S2 Table. Primers used in this study**

| **Primers** | **Sequences (5' to 3')** |
| --- | --- |
| **Sense-*recN*-pro** | CCTTCATATGTTGCTTTGCCTGCGGATTGAAA |
| **Anti-*recN*-pro** | CCTTCTCGAGTCAACTCCTCTGTCCCTGACGGTGA |
| **Sense-*recN*-983** | CCTTGCGGCCGCTAATTCATCCAAAATCCCAACTAAG |
| **Anti-*recN*+1722** | CCTTCTGCAGACTCCTCTGTCCCTGACGGTGATTA |
| **Sense-*dnaA*-693** | CCTTGCGGCCGCGTTGTAGATTTTGTCGCAGCTAGAG |
| **Anti-*dnaA*+1377** | CCTTCTGCAGCTTCGTTTCCATCCAGTTTTCCCCT |
